# Piezosurgery in hemifacial microsomia: a promising exemption from conventional peri-osteotomy suffering

**DOI:** 10.3389/fped.2023.1149710

**Published:** 2023-06-30

**Authors:** Xuetong Wang, Byeong Seop Kim, Ziwei Zhang, Hayson Chenyu Wang, Yan Zhang, Gang Chai

**Affiliations:** Department of Plastic and Reconstructive Surgery, Shanghai Ninth People’s Hospital, Shanghai Jiao Tong University School of Medicine, Shanghai, China

**Keywords:** piezosurgery, osteotomy, mandibular distraction osteogenesis, hemifacial microsomia, intraoperative blood loss

## Abstract

**Introduction:**

Mandibular distraction osteogenesis, a recommended therapy for hemifacial microsomia, has brought much agony because of its traumatic procedures and peri-osteotomy complications. Our study aims to retrospectively compare piezoelectric osteotome with conventional reciprocal bone saw for hemifacial microsomia patients and validate its meliority in operability, surgical risks and patient outcomes.

**Methods:**

All patients included underwent osteotomies conducted by either piezosurgery or bone saw. Information of intraoperative blood loss, operation duration, postoperative pain and complications was collected from patient files, ward round inspections and follow-ups.

**Results:**

Among all 40 patients, 13 underwent piezo-osteotomy. Piezosurgery performed better than conventional reciprocal bone saw in decreasing intraoperative blood loss (*p* < 0.001) and operation duration (*p* = 0.030). No significant difference was found in hospitalization duration, total expenses or complication rates between two groups. There were positive relations between operation duration and intraoperative blood loss (*p* = 0.042), and between hospitalization duration and total expenses (*p* = 0.0096). Postoperative pain scores of both groups declined over time while the piezosurgery group had a statistically significant tendency (*p* = 0.006) to suffer less than the conventional group.

**Discussion:**

Piezosurgery diminishes intraoperative blood loss, operative duration, and postoperative pain, making an alternative to conventional osteotomes to mitigate patients' and families' peri-osteotomy sufferings, and a more humane solution to HFM.

## 1. Introduction

Hemifacial microsomia (HFM), a 1/5,600 incidence congenital disease clinically graded according to Pruzansky–Kaban classification, is mainly featured by unilateral or bilateral facial bony, muscular, and neural hypoplasia with resultant craniofacial asymmetric deformity and occlusal dysfunction (1).

For decades, mandibular distraction osteogenesis (MDO) has been the optimal choice for patients with Pruzansky–Kaban type II and III (2, 3), yet it alleviates and agonizes at the same time—osteotomy, an essential part of distraction osteogenesis, acts as the culprit to a once-reported 4.5% mortality and most other sufferings (4). Though through multiple modifications, MDO has never cast off thorns related to its traumatic procedures (5), namely, pain or complications such as tooth germ injury, vessel injury, nerve injury, and infection (6).

Hence, appeal for a smarter and less invasive strategy arises. Piezosurgery debuted on an operating table in 1975 (7) and since then has acquitted itself well on safety, preciseness, and sensitivity (8, 9). The high-intensity focused ultrasonic apparatus selectively works on mineralized structures, thanks to its vibration frequency of 29 Hz and consequent vaporization of intracellular fluid along with breakage of hydrogen bonds (10, 11). Ultrasound creates a cavitation phenomenon with hemostatic effect on the site, and at the same time little jounce or overheat generated by the micro-vibrations means little interference with surgeons’ manipulation (12). Given this nature, contact with unplanned areas, coagulative necrosis, and soft tissue injuries could largely be avoided in piezosurgery (10, 13, 14), thus making it a promising alternative to conventional osteotomy techniques.

Whereas the implementation of piezosurgery is rarely reported in hemifacial microsomia, neither has it been compared with reciprocal bone saws (15). Our study aims to fill this void by an investigation into differences between them through perspectives including operability, surgical risks, and total hospitalization expenses and validate the meliority of piezoelectric scalpel over bone saw for HFM patients.

## 2. Materials and methods

### 2.1. Patients

A total of 38 patients aged from 2 to 14 years with clear diagnosis of HFM OMENS-Plus (modified orbit, mandible, ear, nerve, and soft tissue system) classification type M2A, M2B, and M3 (16) who have had preoperative and postoperative three-dimensional computerized tomography (CT) scan images as well as good oral hygiene and nutrition are included in this study. All patients underwent an inpatient MDO surgery with admissions to the Plastic and Reconstructive Surgery Department of Shanghai Ninth People's Hospital not earlier than 1 January 2016 and their discharges not later than 9 November 2021, among which 11 had piezosurgery instead of conventional osteotomy based on the patient and parent's decision after full understanding of both choices. Poor compliance, motivation, or follow-ups make the criteria of exclusion.

### 2.2. Ethics

This study has been approved by the ethics committee of Shanghai Ninth People's Hospital (2016-156-T105) and carried out in accordance with the World Medical Association Declaration of Helsinki (2013 amended version). Parents have been informed of the surgical risks before they chose either way of osteotomy and then given their written consent to patient inclusion.

### 2.3. Surgical procedures

Before the surgery, archives, preoperative full cephalometric three-dimensional CT scan (64 slices, SOMATOM Definition Flash 80 kV, Siemens, Berlin, German), and virtual surgical planning were arranged for every patient. Under general anesthesia, surgeries were performed by the same adept team consisting of craniomaxillofacial surgeons, registered nurses, and anesthesiologists.

**Figure 1:**
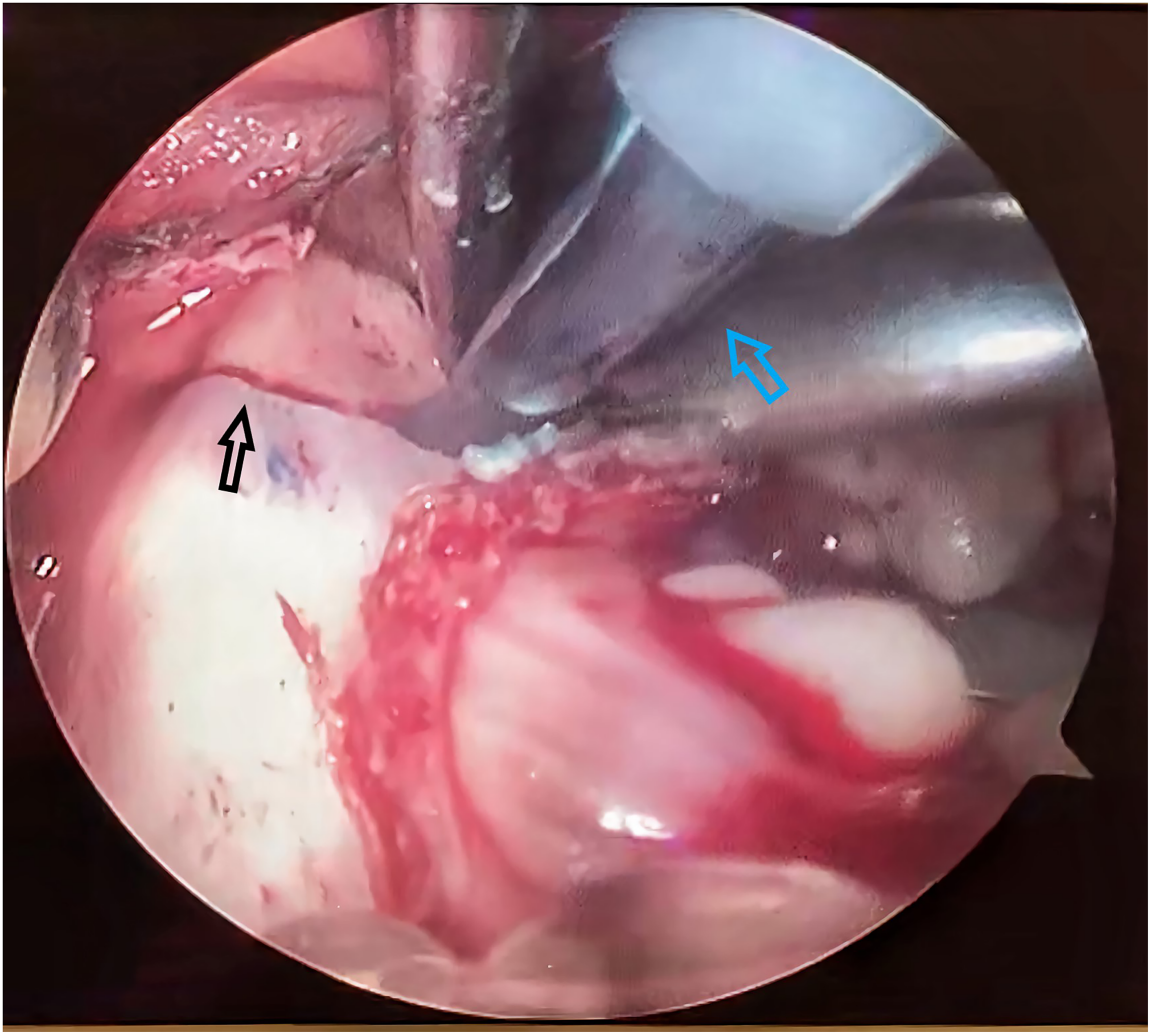
Piezosurgical blade cutting along the osteotomy line, a view under endoscope. The black arrow points to the osteotomy line. The blue arrow points to the piezosurgical blade.

An intrabuccal incision was performed after injection of 1% lidocaine and 1:100,000 epinephrine, along the occlusal line from the first molars to in between the upper and lower teeth. The medial and lateral outer periostea of the mandible were released and carefully managed with a bipolar coagulator to minimize hemorrhage. The surgical guide was fixed onto the ramus under endoscope, between plates of which the osteotomy line was indicated, and then cleaved by either the reciprocal bone saw (Aesculap Power Systems GB130R, Aesculap Inc., Pennsylvania, Unites States, and Bone Saw Blade GJ-38 mm-6 mm, Nantong Robert Medical Technology Co., Jiangsu, China) or piezosurgery (Sonic Control Serrated Aggressive Knife and Sonopet 25 kHz Handpiece, Stryker Instruments, Michigan, United States) from the interior side as shown in Figure 1 and supplemental videos (Supplementary Material S1–S3).

**Figure 2:**
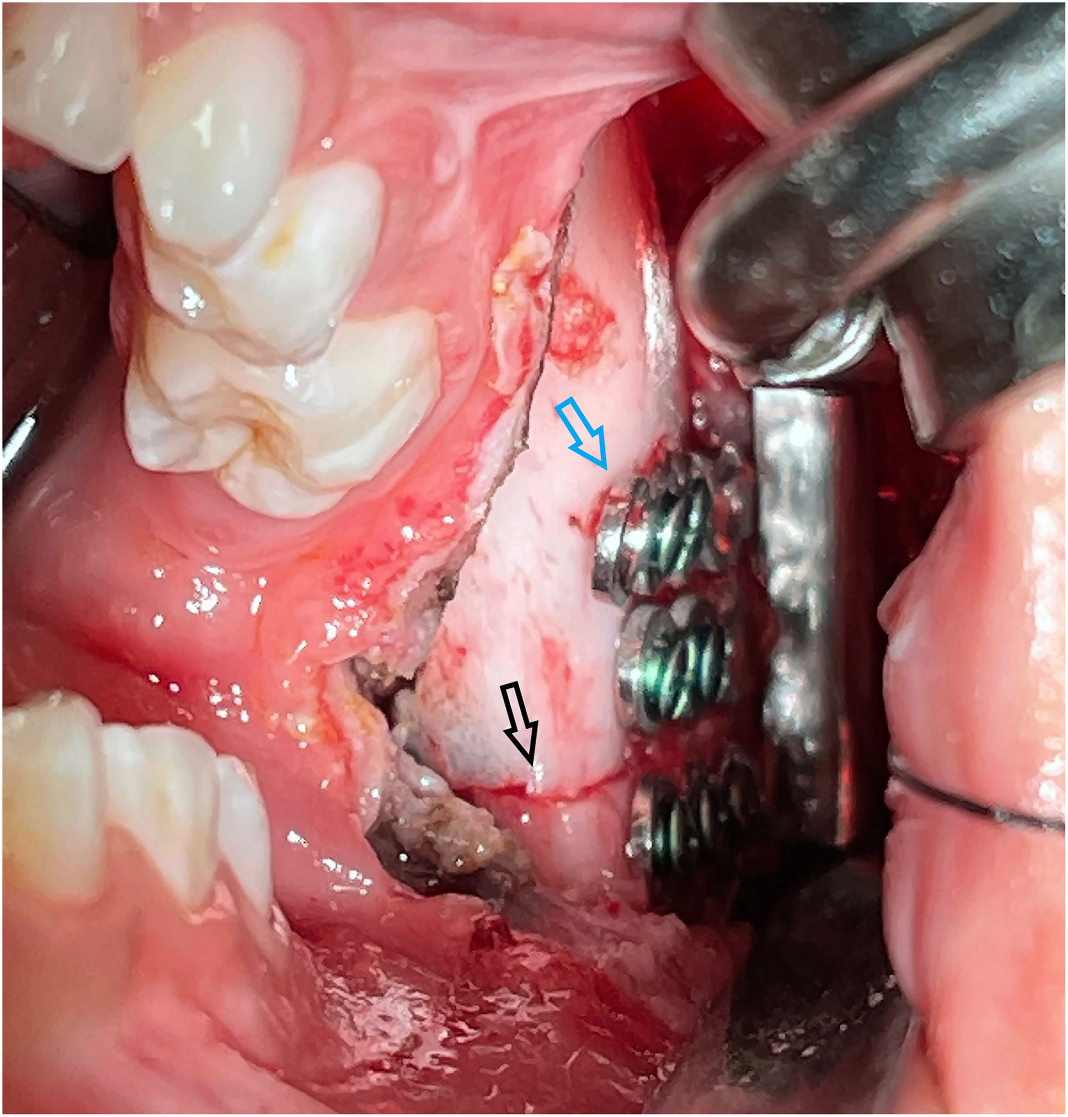
Distractor fixed along the osteotomy line created by piezosurgery, a naked eye view. The black arrow points to the osteotomy line. The blue arrow points to the distractor.

A distractor was installed intraorally and fixed by three screws, respectively, at the proximal end and distal end with protection of the inferior alveolar nerve (Figure 2).

After insertion of a drainage, the oral mucosa was sutured with absorbable Vicryl 5-0 (Ethicon Inc., New Jersey, United States).

Following a 7-day postoperative latency phase, the distraction was initiated and kept at a pace of 1 mm elongation per day. This process was terminated at cephalogram and panoramic radiograph confirmation of overcorrected mandibular midline and followed by the distractor removal surgery.

### 2.4. Data collection and assessments

Intraoperative blood loss and operation duration were accessed from the anesthesia chart of every surgery, while postoperative pain and complications were revealed by subsequent inspections during hospitalization. Patients were asked to point out their pain level on a visual analog scale (VAS) on ward rounds from day 2 to day 5 after surgery. For non-verbal young patients (17), children's behaviors were observed to assess their pain level according to the face, legs, activity, crying, consolability pain scale (FLACC). Facial sensation examination and dressing change were also carried out every day for elimination of complication–infection or lower lip numbness that implied injuries to the inferior alveolar nerve. Hospitalization expenditure information was acquired from the first page of each patient's inpatient record. During regular follow-ups, patients or parents were asked to report any observation of complications till their next admission for distractor removal surgery. The panoramic radiograph at this time would disclose potential injuries to tooth buds.

All data were assessed by IBM SPSS Statistics Software Package, version 20.0 (IBM Corporation, New York, United States). Statistical methods include independent sample *t*-test, chi-square test, correlation analysis, and general linear model. All statistical analyses in this study was given α = 0.05 and statistical significance level at *p* < 0.05.

## 3. Results

Data of the 38 patients aged 2–14 years with diagnosis of HFM OMENS-Plus type M2A, M2B, and M3 were collected. Eleven of them (28.9%) underwent osteotomy conducted by piezosurgical instruments. These patients were defined as the piezosurgery group. The other 27 patients who underwent reciprocal bone saw osteotomy were defined as the conventional group. Other epidemiological data were listed in Table 1. Differences between intraoperative blood loss, operation duration, postoperative pain level, hospitalization duration, and total hospitalization expenses were analyzed by the independent sample *t*-test, while comparison between complication cases of conventional osteotomies and piezosurgery was made by the chi-square test. Relevance between intraoperative blood loss and operation duration or hospitalization duration and total expenses was achieved through the correlation analysis. Postoperative pain scores on different days and their tendency were analyzed by the general linear model.

**Table 1:** Epidemiological data of all patients.

		*N* in piezosurgery group (total = 11)	*N* in conventional group (total = 27)
Age	2–6	3	5
	6–10	4	6
	10–14	4	16
Gender	Male	8	18
	Female	3	9
OMENS-Plus type	M2A	6	12
	M2B	5	14
	M3	0	1

*N*, number of the cases.

The mean amount of intraoperative blood loss was 95.56 ml (SD: 60.98 ml) in the conventional group, 38.18 ml (SD: 17.22 ml) in the piezosurgery group, and 78.95 ml (SD: 58.21 ml) in all patients. There was a distinct difference (*p* < 0.001) between the two groups.

The operation duration with a mean of 129.55 min (SD: 42.039 min) was also significantly lower (*p* = 0.030) in the piezosurgery group compared with a mean of 165.19 min (SD: 43.445 min) in the conventional group. The mean operation duration in all patients was 154.87 min (SD: 45.522 min).

No statistical significant difference was found in hospitalization duration or total expenses between the two groups (Table 2).

**Table 2:** Comparison of intraoperative blood loss, operation duration, hospitalization duration, and total expenses between piezosurgery group and conventional group.

*n*	All patients	Piezosurgery group	Conventional group	*p*
	40	13	27	
Intraoperative blood loss (ml), mean (SD)	78.95 (58.21)	38.18 (17.22)	95.56 (60.98)	<0.001
Operation duration (min), mean (SD)	154.87 (45.52)	129.55 (42.04)	165.19 (43.45)	0.030
Hospitalization duration (days), mean (SD)	7.68 (1.73)	7.55 (1.13)	7.74 (1.93)	0.756
Total expenses (CNY), mean (SD)	58,352.30 (8,988.27)	54,193.00 (7,702.39)	60,046.82 (9,047.10)	0.068

*p* < 0.05 statistical significance; *n*, number of the cases; CNY, China Yuan.

Five patients in the conventional group (18.52%) were reported to have postoperative infection, while none in the piezosurgery group was found with complications (Table 3). Yet this difference was not statistically significant (*p* = 0.126). These patients were found to have stayed shorter in hospital and reported their infections after discharge. The correlation analysis between hospitalization duration and postoperative complications reported an *r* = −0.248 and a unilateral *p* = 0.108.

**Table 3:** Comparison of complications between different groups.

	Postoperative infection	Without complications	Total	Complication rate	*p*
Conventional group, *n*	5	22	27	18.52%	0.126
Piezosurgery group, *n*	0	13	13	0%	
Total, *n*	5	33	40	13.16%	

*n*, number of the cases.

*p* < 0.05 statistical significance; no other complications occurred besides infection.

**Table 4:** Comparison of postoperative pain VAS scores between piezosurgery group and conventional group.

Postoperative pain VAS scores	Piezosurgery group	Conventional group	*p*
Day 2, mean (SD)	4.64 (1.03)	5.89 (1.74)	0.032
Day 3, mean (SD)	4.00 (1.00)	5.67 (1.44)	0.001
Day 4, mean (SD)	4.74 (1.61)	3.73 (0.90)	0.020
Day 5, mean (SD)	3.18 (1.08)	3.81 (1.44)	0.199

*p* < 0.05 statistical significance.

The correlation analysis showed positive relations with medium strength between operation duration and intraoperative blood loss (*r* = 0.331, *p* = 0.042) and between hospitalization duration and total expenses (*r* = 0.415, *p* = 0.0096).

**Figure 3:**
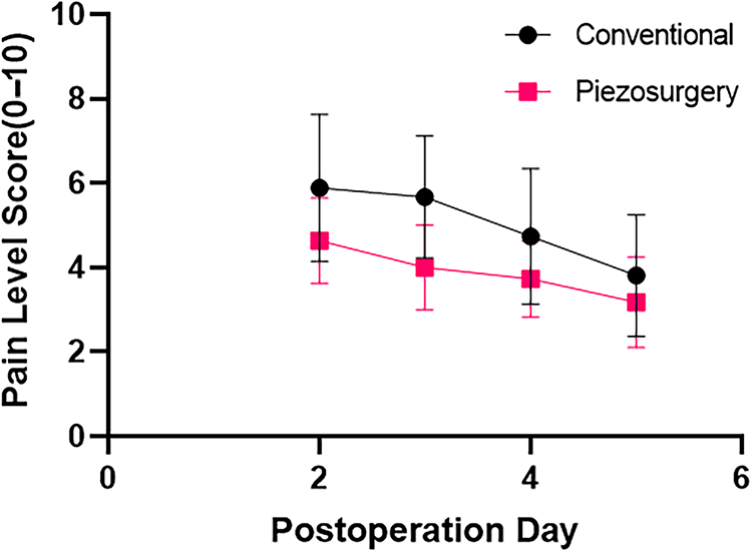
Postoperative pain score change tendency in two groups. The pain level ranges from 0 to 10, 0 represents no pain and 10 represents extreme pain. The black line represents the conventional group, and the red line represents the piezosurgery group.

Distinct differences were found in postoperative pain scores on day 2 (*p* = 0.032) and day 3 (*p* = 0.001) between the two groups. The mean scores were, respectively, 5.89 out of 10 (SD: 1.74) in the conventional group and 4.63 (SD: 1.03) in the piezosurgery group on day 2 and 5.67 (SD: 1.44) in the conventional group compared with 4.00 (SD: 1.00) in the piezosurgery group on day 3. Although postoperative pain scores of both groups were declining over time (Table 4), the patients in the piezosurgery group had a statistically significant tendency (*p* = 0.006) to suffer less than those in the conventional group (Figure 3).

## 4. Discussion

Patients, especially children, with severe HFM (OMENS-Plus classification type M2A, M2B, and M3) are vulnerable to great social pressure owing to their appearance. Yet MDO, the most widely endorsed therapy for these patients, despite its esthetic and auxetic improvements, burdens patients with pain and their family with high medical expenses and large efforts on aftercare attributed to its technical difficulty, complexity, and risks.

In this rapidly developing era, empathetic doctors like us strive to improve MDO from various aspects to lighten patients’ burdens, namely, cephalometric technique of better precision—3D CT, more elaborated preoperative design—virtual modeling, more flexible intraoperative guidance-augmented reality, advanced anesthesiology and nursing science, or even some subtle details such as bipolar coagulation. The newly emerged surgical instrument piezosurgery has been seen as a boon by the orthopedists and therefore gains our preference as an alternative to conventional osteotomy devices.

Piezosurgical device transduces electricity toward mechanical energy and makes the titanium tool bit vibrate at a high-frequency resonance, strong acceleration generated by which allows efficient pulverization, and cutting on target bones, thus distinctly shortening the operation duration. Upon the first contact with bone tissues, the piezoelectric tool bit acts immediately yet purely on the bone in a restricted scale of a few hundred micrometers with density selectivity that switches its work mode to low-frequency vibration on low-density objects, expelling the possibility of wringing, grinding forces, and damages to adjacent tissues. This may explain a smaller dissection field on the interior side of the mandible caused by piezoelectric osteotome when entering through an intrabuccal incision. Conversely, bone saws with a larger range of motion tend to impact soft tissues and, in most cases, penetrate the mandible, causing neurovascular injuries on the lateral side.

A build-in cooling system on the piezoelectric device reduces the heat load, prevents marginal osteonecrosis which could impact the ability of bone regeneration and ultimately the effect of distraction osteogenesis, and at the same time washes away bone debris during the osteotomy to tidy the fracture end and give a clear vision. Piezoelectric scalpels with micro-vibrations are of less tendency to bounce and at greater ease to handle, so when surgeons find out an exposed nerve or vessel, or an excessive entrance of tip close to tooth buds and intend to detour, there would be few interference from scalpels’ own jounce with their delicate grasp of movement directions. In other words, piezosurgery has less dependence on surgeons’ feeling or pre-judgment; instead, it endows with enough time to respond and redress. This precaution against inexperience results in easy manipulation and long-term pedagogical benefit for beginner surgeons. Thermal effect arisen from vast cavitation bubble rupture during piezosurgery working coagulates bleeding vessels with denaturized protein clots in adjacent tissues and decreases intraoperative blood loss to facilitate better convalescence.

Both osteotomy instruments achieve anticipated surgery effects with few noticed differences albeit an extra cost of a few tenths of a millimeter's length on the mandibular ramus on account that the thickness of piezoelectric scalpel is twice that of a bone saw. Though this discrepancy is too subtle to be visualized on CT imaging or with the naked eye, it makes a foremost limitation of this study. The lack of measurements on sectioned bone volume (18) or ultimate distraction length in three-dimensional reconstructed models leads to insufficient surgical result evaluation. This is expected to be covered in a future study. Another limitation is a small sample size due to the low incidence of HFM and a selection bias due to scarce promotion of piezosurgical instruments. Higher procurement costs of piezosurgical instruments compared with conventional ones complained by hospital administrative departments may explain its low popularization from an unexpected view.

## 5. Conclusion

Piezosurgery serves as a next-generation way of osteotomy with unique property that offers definite convenience and support to surgeons. It preserves the outcomes of MDO and simultaneously diminishes intraoperative blood loss, operative duration, postoperative pain, and complications of osteotomy, including soft tissue, neurovascular, and tooth bud injuries. In our outlook, the safer, more efficient, and more approachable alternative to conventional osteotomes, piezosurgery, is able to mitigate patients’ agony and families’ financial and energy drain and open the vista of a more humane solution to HFM.

## Data Availability

The raw data supporting the conclusions of this article will be made available by the authors, without undue reservation.

## References

[1] Birgfeld C Heike C. Craniofacial microsomia. Clin Plast Surg. (2019) 46(2):207–21. 10.1016/j.cps.2018.12.001 30851752

[2] Young A Spinner A. Hemifacial microsomia. Treasure Island, FL: StatPearls Publishing (2021). 32809654

[3] Bogusiak K Puch A Arkuszewski P. Goldenhar syndrome: current perspectives. World J Pediatr. (2017) 13(5):405–15. 10.1007/s12519-017-0048-z 28623555

[4] Dunaway DJ Britto JA Abela C Evans RD Jeelani NUO. Complications of frontofacial advancement. Childs Nerv Syst. (2012) 28(9):1571–6. 10.1007/s00381-012-1804-y 22872275

[5] Sahoo NK Issar Y Thakral A. Mandibular distraction osteogenesis. J Craniofac Surg. (2019) 30(8):e743–6. 10.1097/SCS.0000000000005753 31343587

[6] Winters R Tatum SA. Craniofacial distraction osteogenesis. Facial Plast Surg Clin North Am. (2014) 22(4):653–64. 10.1016/j.fsc.2014.08.003 25444735

[7] Zara F De Sanctis CM Dede FC Bossù M Sfasciotti GL. A split-mouth study comparing piezo electric surgery and traditional rotary burs on impacted third molars in young patients: an intraoperative and postoperative evaluation. Minerva Stomatol. (2020) 69(5):278–85. 10.23736/S0026-4970.20.04349-6 32407060

[8] Liu J Hua C Pan J Han B Tang X. Piezosurgery vs conventional rotary instrument in the third molar surgery: a systematic review and meta-analysis of randomized controlled trials. J Dent Sci. (2018) 13(4):342–9. 10.1016/j.jds.2016.09.006 30895143 PMC6388871

[9] Bertossi D Albanese M Nocini R Mortellaro C Kumar N Nocini PF. Osteotomy in genioplasty by piezosurgery. J Craniofac Surg. (2021) 32(3):e317–21. 10.1097/SCS.0000000000003150 29944550

[10] Küçükkurt S Değerliyurt K. Does piezosurgery decrease patient morbidity in surgically assisted rapid palatal expansion compared with saw and burrs? J Oral Maxillofac Surg. (2020) 78(6):1019.e1–10. 10.1016/j.joms.2020.01.030 32112718

[11] Eggers G Klein J Blank J Hassfeld S. Piezosurgery®: an ultrasound device for cutting bone and its use and limitations in maxillofacial surgery. Br J Oral Maxillofac Surg. (2004) 42(5):451–3. 10.1016/j.bjoms.2004.04.006 15336773

[12] Vercellotti T. Technological characteristics and clinical indications of piezoelectric bone surgery. Minerva Stomatol. (2004) 53(5):207–14. PMID: 15263877

[13] Preti G Martinasso G Peirone B Navone R Manzella C Muzio G Cytokines and growth factors involved in the osseointegration of oral titanium implants positioned using piezoelectric bone surgery versus a drill technique: a pilot study in minipigs. J Periodontol. (2007) 78(4):716–22. 10.1902/jop.2007.060285 17397320

[14] Tosun E Bilgiç M Yildirim B Tüz HH Özer T. Effects of piezoelectric surgery on bone regeneration following distraction osteogenesis of mandible. J Craniofac Surg. (2017) 28(1):74–8. 10.1097/SCS.0000000000003213 27906844

[15] Robiony M Polini F. Piezosurgery: a safe method to perform osteotomies in young children affected by hemifacial microsomia. J Craniofac Surg. (2010) 21(6):1813–5. 10.1097/SCS.0b013e3181f43e03 21119428

[16] Gougoutas AJ Singh DJ Low DW Bartlett SP. Hemifacial microsomia: clinical features and pictographic representations of the OMENS classification system. Plast Reconstr Surg. (2007) 120:112e–3e. 10.1097/01.prs.0000287383.35963.5e 18090735

[17] Merkel S Voepel-Lewis T Malviya S. Pain assessment in infants and young children: the FLACC scale. Am J Nurs. (2002) 102(10):55–8. 10.1097/00000446-200210000-00024 12394307

[18] Hanser T Doliveux R. Microsaw and piezosurgery in harvesting mandibular bone blocks from the retromolar region: a randomized split-mouth prospective clinical trial. Int J Oral Maxillofac Implants. (2018) 33(2):365–72. 10.11607/jomi.4416 29534125

